# ‘You gotta have something to chew on’: perceptions of stress-induced eating and weight gain among office workers in South Korea

**DOI:** 10.1017/S1368980020000890

**Published:** 2021-02

**Authors:** Sohyun Park, Eunju Sung

**Affiliations:** 1 Department of Food Science and Nutrition, College of Natural Science, Hallym University, Chuncheon, Gangwon, Korea; 2 Department of Family Medicine, Kangbuk Samsung Hospital, Sungkyunkwan University School of Medicine, Seoul, Korea

**Keywords:** Qualitative research, Emotional eating, Stress, Dietary behaviours, Obesity, Office workers

## Abstract

**Objective:**

Job-related chronic stress has been discussed as a risk factor for weight change and metabolic disorders. The current study was conducted to understand the situations in which stress-induced eating occurs among office workers and how workers perceive stress to influence their daily eating practices and weight change.

**Design:**

In-depth, one-on-one interviews were conducted with office workers.

**Setting:**

Metropolitan areas in South Korea.

**Participants:**

Twenty-two office workers from thirteen companies participated in the study.

**Results:**

Most participants mentioned that they often felt work-related stress and reported various levels of perceived stress, as measured with open-ended questions. The main sources of work stress were (i) the nature of job characteristics, (ii) performance evaluations and (iii) relationships within the organisation. Participants linked stress with increased food consumption and cravings for sweet, savoury and greasy foods. Many participants emphasised the links between multiple health behaviours and stress. Not only dietary choices but also alcohol consumption, sleeping difficulty and insufficient physical activity were related to coping with work stress and demands. Finally, most participants who perceived work stress believed that their weight gain in adulthood was triggered by work stress.

**Conclusions:**

It is necessary to consider promoting behavioural modifications to support weight management and providing a means for stress management and the minimisation of stress-inducing working environments for workers to maintain or achieve a healthy weight and to prevent chronic disease incidence.

Obesity is a major public health concern in most countries^(1)^, and South Korea is no exception. Recent national data in South Korea showed an increase in obesity prevalence in the working population aged 20–40 years in men (39·0 % of men in their 20s, 46·7 % of men in their 30s and 44·7 % of men in their 40s) and women (18·3 % of women in their 20s and 30s and 25·6 % of women in their 40s)^(2)^. Understanding the various mechanisms that lead to weight gain among working populations is crucial to the national public health agenda.

Exposure to chronic psychological stressors is one possible cause of weight gain during adulthood. Previous research has investigated the links between stress and weight gain^(3)^, and two pathways have been discussed: (i) direct neuroendocrine effects and (ii) indirect effects mediated by adverse health behaviours^(4–6)^. Chronic stress activates the hypothalamic–pituitary–adrenal axis through the release of corticosteroids (cortisol), which may contribute to the accumulation of abdominal fat and lead people to engage in less healthy behaviours, such as consuming more palatable and energy-dense foods^(5)^.

Studies have shown that high levels of stress, as measured and perceived, are related to the modification of long-term and short-term food intake and adiposity^(3)^. In particular, a high level of stress (measured with plasma cortisol) has been associated with increased energy intake and preferences for foods with the characteristics of crispiness and fullness of taste^(7)^, and exposure to stress has been associated with eating in the absence of hunger^(8)^. Longitudinal studies have also shown that a higher stress level predicts a larger increase in BMI^(9–12)^. Most prospective epidemiologic studies show a positive association between stress and weight gain; however, there is an additional evidence that the relationship is not that simple. For example, one cohort study showed that psychosocial work stress was positively associated with future weight gain among obese men, whereas work stress was associated with weight loss among lean men^(13)^.

Possible explanations of the influence of stress on altered eating behaviour and weight change (mainly weight gain) include emotional eating as a stress coping mechanism that often involves eating ‘comfort food’^(14,15)^. Emotional eating is defined as eating in response to emotional cues and has been associated with weight gain and obesity^(16,17)^. Foods with high fat and carbohydrate energetic content may be characterised as comfort foods, and it has been shown that people with emotional eating patterns tend to consume more of these foods^(15)^. Individuals who engage in disordered eating, whether binge eating or night eating (ingesting most of one’s daily energy content at night), generally characterise themselves as chronically stressed^(18)^.

Employees can be exposed to different types of stressors, such as psychosocial and physical stressors. Chronic job-related stress among working populations has been widely discussed in many disciplines, including managerial, physiological and psychological research^(19–23)^. In studying the role of stress on health behaviours, such as dietary practices, psychosocial stressors at work have been the main focus of previous studies and have been found to be an important factor for workers’ weight change^(9,24,25)^. Therefore, in this manuscript, psychosocial stressors were of interest, and work-related stress was defined as ‘the process by which workplace psychological experiences and demands produce both short-term and long-term changes in mental and physical health’, as suggested by Ganster & Rosen^(21)^.

Emotional eating in the work context has not been extensively explored using qualitative study designs. Regarding emotional eating, studies have mainly focused on individuals with eating disorders^(26,27)^, while few studies have used qualitative research methods to understand individuals without eating disorders^(28)^. Qualitative studies have unique strengths in collecting contextual information, which is difficult to capture with quantitative studies^(29)^. Studies on worker stress and the implications of such stress on eating and health outcomes have focused on the correlation or causation between the level of stress and health outcomes, including body adiposity^(13,30–32)^. These studies have primarily used either cross-sectional or longitudinal quantitative research methods. Some qualitative studies have explored work-related stress. Siegel & Sawyer^(33)^ employed a grounded theory approach to study how female workers with pre-diagnosed eating disorders experienced their workplaces. In addition, Bhui *et al.* ^(34)^ explored the perceptions of causes of work stress and effective interventions among employees working in various organisations. However, the literature has not explored how people without clinically diagnosed eating disorders perceive their situations and work-related stress that leads to unhealthy eating choices or other behaviours that are associated with weight gain.

Therefore, the current study used in-depth interviews to examine the various work situations that trigger altered food choices and how workers change their diets and other health behaviours in response to job stress. The current study further explored how workers perceived the link between stress-related eating and weight change.

## Methods

### Participants and recruitment

Detailed information on the data collection methods is described elsewhere^(35)^. In brief, qualitative data were collected from twenty-two office workers with various job characteristics and job grades from twelve companies in a metropolitan area of South Korea. One-on-one, in-depth interviews were selected as the main data collection method due to their flexibility and appropriateness for exploring lived experiences^(29,36)^. Through the use of open-ended questions and probing techniques, personal stories and the meanings the participants attribute to them were collected^(37)^. Data collection occurred between January and June 2014. An initial recruitment flyer was posted on a corporate web bulletin board. The flyer described the aim of the study and the interview process. A total of forty potential participants applied for the interview, and the researchers contacted twenty workers with various job grades, ages and sexes to maximise the variability. Among the twenty workers contacted, eighteen initial volunteers participated in the scheduled interviews, and two could not participate due to schedule conflicts. Four additional participants were recruited through the snowball sampling method^(38)^. These participants, who were under-represented during the initial recruitment period, had higher job grades, were older and had flexible working hours. The research team reviewed the transcripts periodically, and recruitment was stopped when the research team agreed that there had been no new information gathered from the interviews. Participants were rewarded with a $US10 gift certificate to a local store and a pedometer. Participants provided written informed consent before the interviews. The study protocol was approved by the institutional review board of Gangbuk Samsung Hospital (KBSMC 2013-01-142).

### Data collection

All interviews were conducted by the lead author (S.P.), who is female and holds a doctoral degree in public health; the interviews were conducted one on one in place that was convenient for the participants. Most of the interviews occurred in a cafeteria or meeting room at the participant’s workplace during lunch or after work. The interviewer has graduate-level qualitative research training and has performed various qualitative studies, including her doctoral dissertation research. At the time of interviews, the interviewer was a research professor at a medical school. The interviewer did not have a relationship with any of the interviewees prior to the study. During the recruitment process and the completion of the consent forms, participants were informed of the objectives of the research and how the data collected would be used. The duration of each interview was approximately 50–60 min, and the interviews were audio recorded with each participant’s approval. There were no repeated interviews. Field notes were taken during and after the interviews for probing and discussing reflexivity among the research team.

The interviews were prepared, conducted and analysed using the thematic analysis approach. During the development of the interview guide, the social ecological model was used to explore various personal and environmental factors that affected participants’ overall eating practices^(35,39)^, and the guide was piloted with three volunteers before the study. Stress was one of the key factors that was focused on during the interviews and was repeatedly mentioned by most participants. When a participant mentioned stress in an interview, additional probing questions were asked to answer the following research questions:

1. In what types of situations do workers perceive stress in their daily lives?
2. How do workers perceive the relationship between stress and eating behaviours?
3. Are there patterns between stress and other health behaviours that lead to weight change?
4. How do workers perceive the relationship between stress and weight change?

### Data analysis

The recorded interviews were transcribed verbatim by a research assistant and reviewed by the interviewer for accuracy. The analysis included various steps. First, a codebook was developed to code each interview in entirety based on the theoretical framework. The social ecological model was used as a base framework to select the key constructs, and the developed codebook included personal (including emotions and personal stress as triggers for eating or not eating), organisational (including work-related stress) and sociocultural factors^(29,40,41)^. Two authors (S.P. and E.S.) coded the data. A qualitative data management package was used for coding and managing the data (version 6.0, ATLAS.ti; Scientific Software Development).

Second, after the transcripts were coded, the data were summarised in a matrix guided by thematic analysis to identify patterns and repeating themes in the data^(42,43)^. During the interviews, various individual and environmental influences were mentioned by the participants. In a previous article on the current study, mainly social and cultural influences were discussed^(35)^. These influences included a cultural emphasis on harmony and hierarchical work environments. Another main repeated theme was ‘eating when stressed’. Because this theme was mentioned by most participants and was perceived to be one of the greatest influences on workers’ eating behaviours by the participants, special attention was given to further explore the patterns of stress-induced eating (or non-eating) during the data analysis.

Third, based on the aforementioned research questions regarding stress and eating, the interview transcripts were reread several times to identify patterns of stress-inducing situations and determine how stress influenced participants’ eating practices. Because weight gain and weight management, as well as stress-related eating patterns, were also repeatedly mentioned, other health behaviours associated with weight change, such as alcohol consumption and physical activity, were explored to identify patterns among the participants. These processes involved collating codes into potential themes and gathering all the data relevant to each potential theme. The potential themes were reviewed to determine whether they were related to the coded extracts and the entire data set. The refinement and naming of themes were followed. These steps were completed through the identification of quotes and the grouping of the quotes into final themes^(29,42)^. To ensure the credibility of the findings, peer debriefing and member checking were employed while the preliminary data analysis was in progress^(29,38)^.

## Results

### Participants

Twenty-two participants (thirteen men and nine women) aged 27–52 years, with a median age of 35·5 years, participated in the current study. The participants’ job roles varied from professionals (e.g., researcher, programmer and in-house attorney) to more general positions (e.g., customer service representative at a call centre and general administrator). Detailed information on the participants’ job roles, sexes and ages can be found in a previous publication^(35)^ and in Table 1.

**Table 1:** Characteristics and key stress-related eating behaviours among participants

Interviewee	Sex	Age	Job type	Marital status	Perceived work-related stress	Other major sources of stress	Major behaviour for stress management	Stress-related eating	Recent weight change or health problems	PA	Alcohol consumption	Current weight status (self-reported)
A	M	36	IT specialist	Married	Work-related stress is present but not as severe as family issues	Family member’s illness	Exercising	None	Has gained weight due to high energetic intake after starting work	Maintaining regular exercise regime to manage stress	Bing drinking 1–2 times/month	Overweight
B	M	52	General management	Married	Weekly performance report	Issues with teenage children	Drinking moderately	None	None	Maintaining regular exercise regime to maintain health	When younger, drank more for official meetings at work	Overweight
C	F	39	General management	Married	Balancing between work and taking care of children		Attending church activities on weekends	None	Weight change was not mentioned but was diagnosed with thyroid cancer 2 years ago and had a surgery	Tries to exercise regularly to maintain health and manage fatigue	Not often because of children (two boys, school age)	Normal
D	F	30	In-house attorney	Married	Learning a new job (just joined the company)	Weight control issues/late-night binge eating	Nothing in particular; ‘can forget work-related stress during the weekend, and going to church helps’	Eating chocolate while working at the office	Weight has remained steady during adulthood but has always been obsessed with weight control; has a fear of gaining weight	No regular PA	Social drinking	Normal
E	F	32	Reviewer of insurance contract	Single	Frequent arguments with insurance salespeople while reviewing insurance contracts		Eating more than usual, sleeping a lot, walking a lot during weekends	Late-night snacking on chips and ice cream, snacking on sweets during work; ‘I need to have something to chew on’	Gained weight due to lack of exercise during week and snacking on sweets	Only on the weekend (mostly walking)	No drinking	Normal
F	M	39	IT specialist	Married	Low sleep quality due to work-related calls at night (network emergencies)		Eating more than usual	Having cravings for foods; ‘finished two bowls of steamed rice’	Gained weight gradually after marriage and staring the current job	No regular PA (spending more time with children is a priority)	1–2 times/week	Overweight
G	M	39	IT specialist	Married	Excessive workload		Eating more than usual	Late-night snacking due to long working hours and work stress	Gained weight since started working	Started to do PA regularly in the morning recently (could not do PA when started the current position due to heavy workload)	Does not enjoy drinking but has social events with drinking every 2 weeks	Overweight
H	F	33	Customer service representative	Single	People stress while managing rude customers		Eating more than usual	Binge eating sweets, carbohydrates and spicy foods; drinking instant coffee with sugar and cream; drinking sugar-sweetened beverages while on the phone with customers; ‘Drinking sweet instant coffee gets you through a stress-filled work day, especially at 3–4 in the afternoon’	None	No regular PA	No drinking	Normal
I	F	30	Engineer	Married	Social stress from team leaders		Eating more than usual	Binge eating sweets, carbohydrates, spicy foods and greasy foods until I feel like vomiting	Lost 7 kg when first joined the company due to extreme social stress at work and now has regained 9 kg total	Trying to do stretching and walking regularly	Social drinking when hanging out with colleagues and friends	Normal (but diagnosed as overweight due to high body fat percentage)
J	M	37	General management	Married	Change in work responsibilities		Drinking alcohol and smoking cigarettes	Nibbling on snacks after putting the children to sleep, just to relax and relieve stress	Gained 5–7 kg due to drinking and eating at night after joining the company	Trying to do regular PA but not easy because of childcare	Social drinking	Normal (as a result of recent weight control, but used to be overweight)
K	M	33	Sales	Married	Meeting sales quotas		Exercising, eating sweets, smoking cigarettes	Drinking hurriedly and eating quickly; having cravings for sweets	Has maintained weight	Maintaining regular exercise regime to increase work efficiency	Frequent work-related dinners with alcoholic drinks	Overweight
L	F	34	IT specialist	Single	Work-related stress is not severe	Weight control due to family history of obesity	Eating more than usual, especially sweets; running	Having cravings for sweets	Lost a few kilograms since joining the company, not related to stress	Engaging in brisk walking or running for 1 h/d for weight management	Social drinking	Overweight
M	F	29	Sales planning	Single	Excessive workload and social stress		Exercising, having one drink alone at home, playing the piano	Losing appetite when stressed and reducing the amount of food eaten	No change in weight but has stress-related enteritis a few times per year	Maintaining a regular exercise regime 3–4 times per week	Social drinking	Normal
N	M	29	Researcher	Single	Meeting deadlines to present results		Exercising	Eating more when stressed	Gains a few kilograms when work is intense and loses the weight when work is slow	Maintaining a regular exercise regime for weight control	Social drinking	Normal (as a result of recent weight control, but used to be overweight)
O	M	35	Semiconductor production planner	Married	Excessive workload; returning home late almost every night (21.00–22.00 hours)		Hiking on Sundays, smoking cigarettes	Late-night dinner/snacking due to long working hours and work stress	Gained 6 kg after marriage and then another 12 kg recently	Going hiking only on Saturday mornings because of childcare and busy work	Work-related dinners with drinks; frequent consumption of 1–2 drinks per night at home with late dinners	Obese
P	M	32	Researcher	Married	Meeting deadlines to present results; excessive workload and too few researchers		Exercising	None	Has gained 8 kg since starting the current job (more energetic intake and less expenditure)	Trying to do regular PA for weight control	Social drinking	Overweight
Q	F	27	Producer	Single	Social stress; having to stay late in the office to get work done		Eating more than usual, hanging out with friends	Having more foods than drinks when hanging out with friends because of an inability to handle alcohol	Gained 2–3 kg when first joined the company due to frequent eating/drinking at work gatherings	No regular PA	Social drinking	Normal (but wants to slim down her lower body)
R	M	43	General management	Married	Excessive workload		Drinking alcohol	Drinking alcohol due to stress, which is followed by a stomach ache and in turn leads to consuming more foods	Gained a few kilograms, not because of stress but because of less PA	Maintaining regular exercise regime for 1 h/d	More alcohol consumption when stressed	Overweight
S	F	38	Food and beverage business developer	Married	Excessive workload; having to stay late almost every night (23.00 pm–24.00 hours) during intense projects		Eating more than usual, especially greasy, heavy, meat-based foods and sweets; drinking coffee	Overeating, especially when staying late at work (meat-based foods, cakes, coffees, etc.)	Gained 6–7 kg during a 2-month period of intense projects due to late-night heavy dinners and a lack of exercise. Trying to lose the weight when the project is over; was diagnosed with hyperlipidaemia 2 years ago	Maintaining regular exercise regime for 1 h/d when work is not too intense	No drinking	Normal
T	M	41	IT specialist	Married	Balancing between work and family	Taking care of issues of extended family members	Eating more than usual	Eating to help relieve stress and make him happy	Has gained 7–9 kg since married	No regular PA	No drinking for religious reasons	Overweight
U	M	47	General management	Married	Leading the team to earn good performance reviews and a promotion to an executive level (due to severe stress, having trouble falling asleep at night)		Drinking alcohol before sleeping at night	When drinking at night, eating fried chicken or other finger foods	Has gained 6–7 kg since started working	No regular PA; sometimes practicing golf indoors on weekends	Regular alcohol consumption at home before going to bed	Borderline between overweight and obese
W	M	45	General management	Married	Work-related stress is not severe; ‘just get stressed from time to time as other people would do’	Marital problems	Eating more than usual, drinking more often	Eating more than usual	Has gained 8 kg since started to work; not only because of stress but also because of a lack of exercise, and increased alcohol intake when work is busy and stressful	Maintaining a regular exercise regime for 1 h/d	More alcohol consumption when stressed	Overweight

PA, physical activity; IT, Information Technology.

The qualitative findings are presented in the following order: (i) circumstances in which office workers felt stressed in their daily working lives, (ii) the impact of perceived stress on eating behaviours, (iii) participants’ descriptions of stress and other health behaviours that led to weight change and (iv) participants’ perceptions of the relationship between stress and weight gain. The main themes and sub-themes are summarised in Table 2.

**Table 2:** Themes of stress-related eating behaviours and other health behaviours related to weight change

Theme 1	Situations when employees felt stressed at work
Subtheme 1·1	The nature of job characteristics
Subtheme 1·2	Excessive workload and long working hours
Subtheme 1·3	Performance evaluations
Subtheme 1·4	Relationships with people within the organisation
Theme 2	Stress and its relationships with eating behaviours
Subtheme 2·1	Changes in the amount of food eaten
Subtheme 2·2	Changes in the types of flavours people have cravings for
Theme 3	Stress and other health behaviours related to weight change
Subtheme 3·1	Drinking alcohol with energy-dense foods
Subtheme 3·2	Lack of physical activity
Subtheme 3·3	Difficulty sleeping

### Theme 1. Situations in which employees felt stressed at work

All the participants mentioned that they ‘often feel work-related stress’ and reported various levels of perceived stress. In addition, some mentioned other sources of stress, which mainly included ‘family issues’.

In terms of work-related stress, four sources of stress emerged from the analysis: (i) the nature of job characteristics, (ii) excessive workload and the consequential long working hours, (iii) performance evaluations and (iv) relationships with people within the organisation. A wide range of stress due to the nature of the job was expressed by many participants; the following quotations illustrate some of the circumstances.

One participant was a female worker in her early 30s whose main job was to review the details of contracts with freelancer insurance salespeople and approve them. While doing her job, she had to manage various complaints from the salespeople, which increased her stress level (quote no. 1 in Table 3). Another example showed that when the participants’ jobs involved fulfilling a sales quota or closing large deals within a certain timeline, workers perceived stress (quotes no. 2 and no. 3 in Table 3).

**Table 3:** Selected quotations from the interviews

Quote no.	Quotes
1	‘When I talk to freelancer salespeople, quite often, they get angry or act out when things don’t go their way. They think that I am controlling them on behalf of the company… I am just trying to follow the corporate protocol. But **I** cannot act out like them. So, in the process of bearing all the complaints and acting out, the stress level goes up, and it builds up’. (E)
2	‘My team members tend to have a high level of stress…because we have the pressure all the time to meet sales quotas and close deals’. (K)
3	‘My workload is getting heavy and includes small contracts with various institutions for food and beverage businesses…such as food courts at shopping malls or snack bars at museums. When my team is preparing for an open bid, that means many, many working nights. … I feel like my life expectancy is getting shorter when we are in the middle of the projects’. (S)
4	‘Like at college, we get an annual performance review with letter grades: A, B, C, and D. I would be less stressed if I could be satisfied with a B+. … I wouldn’t try harder to get an A+. But here at the company, an A+ means more money, and a D means a pay cut. So, it’s hard not to be stressed about the evaluation.’ (G)
5	‘When I first joined this firm…like 6 years ago, the head of my department gave me such a hard time. It was super stressful… even my appetite was completely gone’. (F)
6	‘Working in the organization is stressful. People stress…? The work itself is interesting, and I enjoy it. But the amount of work is a lot, and the relationships with people are difficult’. (M)
7	‘My only pleasure is eating when I am stressed. That gives joy to my body. … I think the desire to eat food is the most important basic desire that human beings have’. (G)
8	‘When I am stressed, I eat a lot… like **a lot.** Not just a lot of rice; I eat rice, bread, and rice cake…anything that is available’. (H)
9	‘When I was under **extreme** stress, I lost my appetite…entirely. I couldn’t eat at all. I felt hungry but lost my desire to eat. (Because of my boss) I couldn’t eat at work, then ate a little when I got home. … From my past experience, I learned that with moderate level of stress, I tend to binge eat, but under extreme stress, I can’t eat’. (I)
10	‘When I first joined this firm, I had so much work. So, it was very common to eat dinner at work and go home very late. By the time I got home, I was starving. Because of the work demand and stress that was associated with it and hunger, of course, I ate all sorts of things that I saw at home. Fruit, chocolate candies, bread…everything’. (G)
11	‘When stress builds up, I look for something to eat unconsciously. I gotta have something to chew on. Mostly, something sweet. You know, when you have something sweet, your serotonin level goes up… it’s serotonin, right?… and you become happy. (Laughs)’ (E)
12	‘I think I go out for drinks more often when I am stressed out. More drinking outings with friends and colleagues’. (R)
13	‘Since I became the head of the team, I feel like I have to drink at least one can of beer before falling asleep (because of stress). I also enjoy sharing some fried chicken with my children while I have beer’. (U)
14	‘…I think satisfying my appetite is very important to manage stress and to feel happy. I don’t know about others, but to me, eating is the biggest joy. … To achieve self-satisfaction and manage stress, some people might pursue other activities, such as playing sports. I, too, enjoy swimming and skiing but don’t have time to do sports on workdays. Given the limited free time, I found eating is the only option’. (G)
15	‘When I am in the middle of an intense project, I notice that my health collapses. … During the project period, my body fat percentage goes up, especially visceral fat. I try to slim down during peaceful periods because I know it will go bad again once I start a project again’. (S)
16	‘Since I started this job, I have gained weight gradually, mainly due to work stress and late-night snacking because of working late. And I have no time to work out. It really is a vicious cycle’. (G)

A second source of stress was heavy workloads and the consequential long working hours. The nature of participants’ jobs could include long working hours, as illustrated in quote no. 3; however, simply working late for many nights due to a heavy workload could be stressful. A producer (Q) said that she had to finish editing a weekly programme before the show date, which meant that she needed to stay late at work at least 2–3 nights before the show date every week. Another employee (O) also mentioned that, as he was responsible for more duties after being promoted to manager, he could not have any personal time during workdays and usually arrived home at 21.00–22.00 hours.

A third type of work-related stress was associated with periodic performance evaluations (quote no. 4 in Table 3). Employees with relatively lower level positions perceived stress related to their individual evaluation results, and the pressure was even higher for team leaders. Poor team evaluations equated to a negative outlook for leaders’ future promotions and an unpromising career path for younger team members.

Stress levels seemed to be the highest for employees in higher level positions who were expecting a promotion to an executive-level position (three participants (B, R and U) were in this category). One team leader in his late 40s spent most of his interview explaining how much stress he had to manage on a daily basis. For him, a low rating on a performance review would mean that he would not achieve an executive-level position in future promotion opportunities (only 5 % of team leaders were promoted to executive officer positions, and the remaining were expected to leave the company). His performance evaluations also affected his team members’ career prospects. He stated, ‘I feel this big responsibility and pressure that I should show vision and passion for my team members’ careers. My evaluation is not just for me, it’s for the team’.

A final source of job-related stress was relationships with people within organisations or customers. In particular, relatively young female workers in their 20s and 30s felt stressed due to their teams and departments. Since most employees worked as part of a team and in a department, different communication styles and working styles often triggered stress (quotes no. 5 and no. 6 in Table 3). One participant mentioned that the head of the department seemed to be angry with her all the time for no specific reason, which made her work situation ‘so difficult and stressful’ (quote no. 5 in Table 3).

### Theme 2. Stress and its relationships with eating behaviours

Participants linked stress with (i) the amount of food they ate and (ii) the types of flavours they craved when under stress. In terms of the amount of food participants ate, two different patterns of eating behaviours in response to stressful situations emerged: the participants either ate more or completely lost their appetites under stress. Except for one female participant, all the participants mentioned that they tended to eat more under stress. When one participant was asked how he changed his food intake when stressed, he responded, ‘Of course, eat a lot! Don’t you do that?’ (Interviewee U). Additional quotations in Table 3 (quotes no. 7 and no. 8) illustrate the situations when participants ate more when stressed.

Only one young female participant said that she ‘completely lost [her] appetite when stressed’. She also added that she was ‘the opposite of the most people around her’ (Interviewee M). Another female worker shared her experience of how the intensity of stress affected her appetite. When she was exposed to extreme stress, she lost her appetite entirely, but when she was exposed to mild stress, she tended to binge eat (quote no. 9 in Table 3).

As mentioned earlier, work-related stress was often associated with long working hours. When workers stayed late at work, by the time they arrived home, they were hungry. Many participants mentioned that they ate late at night because of their long working hours and stress, and most of them could not separate these two factors (quote no. 10 in Table 3).

Participants also mentioned the types of flavours that they craved when stressed; mostly, they craved something sweet, salty, greasy or spicy. Workers explained that they had cravings for strong flavours (quote no. 11 in Table 3). During the interview, one female worker said that she was meeting a co-worker for barbecue after the interview. She added that her coworker ‘was under extreme stress due to a bad review on an annual performance evaluation’ (I). Another female worker said that she made trips to convenience stores when stressed at work. Usually, she would look for chocolates and candies. She acknowledged that snacking helped improve her mood (Q).

### Theme 3. Stress and other health behaviours related to weight change: alcohol consumption, insufficient physical activity and difficulty sleeping

A large proportion of the participants mentioned that they drank alcoholic beverages more frequently when they perceived stress. In addition, when drinking alcoholic beverages, participants enjoyed finger foods or delivery food, which was usually energy dense (quotes no. 12 and no. 13 in Table 3).

As mentioned above, many participants perceived links between multiple health behaviours and stress. Not only dietary choices but also insufficient physical activity were related to stress and work demand. As presented in Table 1, some participants successfully made a habit of exercising to manage stress, whereas some participants found it difficult to have time for physical activity. A lack of physical activity led to more eating because the participants thought they had no other options to manage stress (quote no. 14 in Table 3).

Finally, some participants linked work stress to difficulty sleeping. One participant explicitly mentioned that her sleep quality was very low due to work stress (participant M), which led to decreased appetite. Another participant had difficulty falling asleep because of work stress and developed a habit of having a can of beer every night to fall sleep, sleeping barely 4 h per night (participant U).

### Theme 4. Perception of the relationship between stress and weight gain

Among the twenty-two participants, two mentioned their past experiences of losing their appetite and losing weight due to work stress. Meanwhile, more than half of the participants (fifteen participants of twenty-two) explicitly mentioned that they had gained weight since they started working, which was mainly due to additional energetic intake and less physical activity (quotes no. 15 and no. 16 in Table 3). Various reasons contributed to this positive energy balance. The major reasons mentioned by workers were stress-induced eating and alcohol consumption as well as late-night snacks or dinners due to long working hours and/or stress.

In addition to weight gain, many participants mentioned exacerbated health conditions associated with work stress, including recurrent episodes of enteritis, development of jaw pain, sleeping difficulty, hyperlipidaemia and fatty liver. One worker mentioned that he thought it was very common among workers to have such health concerns as his, and when the interviewer asked why he thought so, he answered without hesitation, ‘Of course, work is stressful’.

## Discussion

Using in-depth interviews with office workers, we observed that the majority of workers acknowledged the presence of stress-induced eating behaviours characterised by increased energy intake from food and alcoholic beverages and cravings for sweet, savoury and fatty foods. Some participants lost weight due to extreme job stress; however, more than half of the participants believed that their weight gain in adulthood was related to work-related stress and their work schedules. The patterns of job stress and stress-induced eating behaviours described by the participants corroborate the findings of experimental and observational studies in the literature.

In previous experimental studies, for example, after being exposed to acute stress, participants increased their intake of energy and sweet snacks^(8)^ and felt a ‘wanting’ to consume sweet and savoury food, additional energy and carbohydrates and fat^(44)^. Observational studies have also shown that stress-driven eaters tend to eat high-fat and sweet foods (e.g. sausages, hamburgers and pizza and chocolate) and drink alcohol more frequently than other people^(16,17)^.

Such altered eating behaviours occur when individuals feel stressed, and explanations of these behaviours have been proposed in hypotheses about various physiological mechanisms, for example, the ‘comfort food’ hypothesis^(15,45)^. This hypothesis suggests that chronic stress activates the hypothalamic–pituitary–adrenal axis and further increases glucocorticoid and insulin levels. The increases in glucocorticoid and insulin levels trigger cravings for energy-rich meals. The ingestion of palatable food reduces the negative effects of stress by suppressing corticotropin-releasing factor and by stimulating the pleasure-associated anterior nucleus accumbens shell^(46–48)^.

However, altered food choices in response to stressors are not identical for all individuals. Evidence has shown that most individuals tend to change their eating behaviour under stress^(45)^; however, the direction of change in energy intake varies based on individuals’ eating behaviour profiles and personality phenotypes^(49)^. Other studies have also suggested that the impact of stress on eating depends on (i) the type of stressor and (ii) individual physiological and psychological differences^(50)^. Measuring the exact quantity of food intake under different types and levels of stress and other individual confounders was beyond the scope of this qualitative study. Nonetheless, an observation is that the emphasis on the role of stress in food choices varied among participants during the interviews, and two participants shared their experiences of completely losing their appetites when their work stress levels were too intense.

As mentioned in the results, many participants engaged in late-night eating due to long working hours and work stress, which were difficult to separate. These altered meal times have been suggested to be a cause of circadian rhythm disruption and changes in appetite and energy-regulating hormones, which further impairs metabolic function^(51)^. One study conducted among college students revealed that there was a significant association between perceived stress and night eating syndrome, which was characterised by recurrent episodes of eating a large amount of food in the evening^(52)^. Night eating syndrome has also been associated with a higher risk of psychopathology, including mood, anxiety and sleep problems^(53)^. It is not clear if the participants of the current study, who described their late-night eating habits, can be classified as having night eating syndrome based on the current qualitative data. However, stress, sleep problems and altered meal times are all closely related and contributing factors to weight gain^(53)^.

Regarding sleep problems, it has been well documented that chronic stress is prospectively associated with sleep disturbance^(54,55)^. Furthermore, it is also known that sleep has an influence on eating behaviours. Specifically, short sleep duration, poor sleep quality and late bedtimes are all related to increased food intake, poor diet quality and the preference for energy-rich foods, which are all linked to excess body weight^(56)^. Studies have also shown that poor sleep is associated with elevated emotional eating, and short sleep duration may cause stress and elevated food consumption in emotional eaters^(57)^. These phenomena were also observed among office workers in the current study.

Although the majority of the participants experienced job stress and perceived an influence of job stress on their increased energy intake and weight gain, the mechanisms behind the impact of job stress on weight change may be more complex^(9,25,58)^. Regarding stress and prospective adiposity, gender and baseline BMI may play a critical role. A review by Torres & Nowson^(5)^ noted that longitudinal studies have suggested the existence of gender differences in the relationship between stress and weight gain. They reported that chronic life stress appeared to have a causal effect on weight gain and that the possible effect appeared to be greater among men. In addition, the Whitehall study revealed that psychosocial work stress was linked to prospective weight gain among obese men but was associated with weight loss among lean men. In addition, bidirectional effects of job strain on BMI were not observed among women^(13)^. Another study used the Nurses’ Health Study data and showed that baseline BMI was a key factor that determined how job strain affected prospective BMI change. The authors concluded that women with higher BMI may be more vulnerable to BMI gain when exposed to chronic work stress^(30)^.

This vulnerability may be due to individual physiological and psychological differences, as suggested by previous studies^(14,49,50)^. Specifically, higher BMI has been positively associated with the frequency of emotional eating, and emotional eaters have been shown to be at risk for future weight gain^(14)^. In the current study, we found no significant association between BMI and the level of stress-induced eating behaviours. Among the fifteen overweight and obese participants (including participants with a history of overweight), twelve participants reported some level of stress-induced eating behaviours. Among the seven participants with normal weight, five participants reported altered eating choices when stressed.

In addition, the current study showed that stress affected both female and male participants in similar ways (ten of thirteen men and seven of nine women reported some degree of stress-induced eating). This finding is different from that of a study by Bennett *et al.* ^(28)^ on college students. Among college students, female students responded to school stress by increasing their food consumption, while male students reported decreased consumption when stressed. The effect of BMI and gender on stress-induced eating and consequential weight change among working populations should be confirmed using a quantitative research design.

The study results may suggest the importance of stress management in obesity programmes targeting office workers. As shown in Table 1, the majority of the workers, especially male workers, were categorised as overweight or obese, and many of them acknowledged that their unhealthy eating behaviours when stressed led to weight gain. Some of the workers engaged in healthier activities to manage stress, such as playing musical instruments, attending religious institutions, or participating in regular physical activities, whereas others stated that eating was the easiest and quickest solution given their limited time. Because most of the participants in the current study had long working hours, time scarcity may be a substantial obstacle to engaging in healthier activities for stress management. Notably, multiple health behaviours that affected weight change were clustered among the participants. Difficulty sleeping and physical inactivity were the main behaviours discussed during the interviews. Therefore, identifying strategies to incorporate multiple healthier activities (physical and mental) for stress management among workers within work settings is crucial.

Similarly, a review by Geiker *et al.* ^(3)^ emphasised the importance of stress management in implementing successful weight management programmes. A high stress level and low mood interfered with successful weight management programmes and increased the risk of dropout. These authors stressed that programmes that added stress management to an individual’s lifestyle were more effective in achieving weight loss targets than programmes with a diet component only.

In addition, in one study, thirty-four Greek overweight and obese women in a weight loss trial received stress management components that included progressive muscle relaxation and diaphragmatic breathing. These women showed more weight loss results than the control group, which was given nutrition consultation only^(59)^. Another study with forty-four African-American women showed similar results: a greater percentage of weight loss was achieved when participants received stress management strategies along with lifestyle interventions^(60)^. One intervention study with a larger sample of participants also found that a lower baseline stress level predicted a larger reduction in weight after the completion of a lifestyle intervention, but the intervention itself did not include a stress management programme^(61)^. These previous intervention studies all suggested the role of stress in achieving or maintaining healthy weight.

The organisational change efforts are difficult to implement and fail easily^(62)^. In addition, most workplace health promotion programmes target individual behaviour, and supportive organisational change has been limited^(63)^. Despite these difficulties, the current study highlighted the important role of organisational-level work stress interventions, given the high prevalence of stress-related unhealthy behaviours and perceived weight gain that are associated with workplace stress. The organisers of future larger-scale, longer-term workplace obesity prevention programmes should consider including both individual-targeted strategies and work stress management components at the organisational level.

The current study employed in-depth interviews as the main data collection method and was not able to examine various factors that might modify the impact of job stress, eating behaviours and obesity. According to other research, these factors include gender, baseline BMI and different job types (manual and non-manual)^(32)^. The participants in the current study were all office workers with college degrees, which limited the scope of analysis to a relatively homogenous population in terms of socio-economic status and work environment.

Because the interviews were conducted with volunteers, the participants may have been more weight- and health conscious than the general population. The majority of the participants were concerned about gaining weight or being overweight. Therefore, the study results may under-represent individuals who are not concerned about their dietary behaviours or current weight status. Further studies targeting individuals with various job types and low BMI who are not concerned with their current weight status could help provide a full picture of workers’ perceptions of job stress and the influence of job stress on their eating behaviours and weight change.

## Conclusion

Workers are exposed to daily work-related stress, and the manner of coping with daily stress is critical in maintaining health and well-being. Coping with stress through unhealthy eating behaviours was prevalent among the participants in the current study. As individuals increase in age from their 20s to their 40s, greater work and family responsibilities can be expected. Because obesity prevalence increases throughout adulthood, it is critical to understand the role of job stress in obesity incidence among working populations. Based on the findings, multidisciplinary workplace interventions for stress management skills and the modification of health behaviours are suggested as a satisfactory strategy to prevent weight gain and related health problems.
